# Identification of Dietetically Absorbed Rapeseed (*Brassica campestris* L.) Bee Pollen MicroRNAs in Serum of Mice

**DOI:** 10.1155/2016/5413849

**Published:** 2016-08-15

**Authors:** Xuan Chen, Guan-hai Dai, Ze-ming Ren, Ye-ling Tong, Feng Yang, Yong-qiang Zhu

**Affiliations:** Institute of Basic Medicine, Zhejiang Academy of Traditional Chinese Medicine, Hangzhou 310007, China

## Abstract

MicroRNAs (miRNAs) are a class of small noncoding RNA that, through mediating posttranscriptional gene regulation, play a critical role in nearly all biological processes. Over the last decade it has become apparent that plant miRNAs may serve as a novel functional component of food with therapeutic effects including anti-influenza and antitumor. Rapeseed bee pollen has good properties in enhancing immune function as well as preventing and treating disease. In this study, we identified the exogenous miRNAs from rapeseed bee pollen in mice blood using RNA-seq technology. We found that miR-166a was the most highly enriched exogenous plant miRNAs in the blood of mice fed with rapeseed bee pollen, followed by miR-159. Subsequently, RT-qPCR results confirmed that these two miRNAs also can be detected in rapeseed bee pollen. Our results suggested that food-derived exogenous miRNAs from rapeseed bee pollen could be absorbed in mice and the abundance of exogenous miRNAs in mouse blood is dependent on their original levels in the rapeseed bee pollen.

## 1. Introduction

MicroRNAs (miRNAs) are a class of small noncoding RNA that mediate posttranscriptional gene regulation by promoting cleavage or inhibiting translation of the target mRNA in plants or animals and play a critical role in nearly all biological processes, including metabolism and immune functions [1–3].

Recent studies suggest that plant miRNA may serve as a novel functional component of food which makes a critical contribution to maintaining and shaping animal body structure and function [4]. In 2012, a pilot study found that plant miRNAs from the diet will be absorbed by cells of the mammalian digestive tract and then packaged into microvesicles [4]. The microvesicles protect plant miRNAs from degradation and deliver them via the bloodstream to a variety of tissues (liver, kidney, heart, and brain), in which they will regulate cell gene expression [4]. Using the next-generation sequencing technology, Wang et al. demonstrated that human plasma contains a wide range of RNA from many exogenous species, including bacteria, fungi, and foods such as corn, rice, soybean, tomato, and grape [5]. Lukasik and Zielenkiewicz performed a bioinformatics analysis of publicly available raw data from studies on miRNAs composition in human and porcine breast milk exosomes to identify the fraction of food-derived miRNAs, and 35 and 17 miRNA species were identified, respectively [6]. miR-172 is the most highly enriched miRNA in* Brassica oleracea*, and after feeding mice with* Brassica oleracea*, miR-172 was found in the stomach, intestine, serum/blood, spleen, liver, kidney, and feces of mice [7].

Studies have shown that food-derived plant miRNAs have immunomodulating effects such as anti-influenza virus and antitumor [8, 9]. Zhou et al. found the first active miRNA in traditional Chinese medicine named miR-2911, a honeysuckle- (HS-) encoded atypical miRNA, that directly targets various influenza A viruses (IAVs) [8]. Subsequently, Yang et al. reported that miR-2911 levels fluctuated among various herbs. Feeding these different herb-based diets to the mice leads to different miR-2911 levels in the sera and urine which is associated with dietary intake levels [10]. Mlotshwa and others synthesized 3 tumor suppressor miRNAs (*miR-34a, miR-143, and miR-145*) with a characteristic of plant miRNA [11], and they reported that oral administration of the cocktail reduced tumor burden in well-established Apc*Min/+ *mouse model of colon cancer [12]. Furthermore, Western donor sera contained the plant miRNA miR159, whose abundance in the serum was inversely correlated with breast cancer incidence and progression in patients, and they demonstrated for the first time that a plant miRNA can inhibit cancer growth in mammals* in vivo* and* in vitro *[9].

Rapeseed (*Brassica campestris *L.) pollen is microgametophytes of rape. Bee-collected rapeseed pollen is widely used in food and healthy products [13].* In vivo* and* in vitro* experiments demonstrated that the immune modulating effects by bee pollen might be attributed to its prevention and treatment for diseases [13–15]. Besides, RNA is rich in rapeseed bee pollen [16]. Nevertheless, whether miRNAs in rapeseed bee pollen could be absorbed by animals remains unclear.

In this study, ICR mice were fed with rapeseed bee pollen, and then plant miRNAs including rapeseed miRNAs in mice blood were detected using next-generation sequencing technology.

## 2. Materials and Methods

### 2.1. Rapeseed Bee Pollen

The rapeseed bee pollen was bought from Bee Research Institute of Anhui Agriculture University. The implementation of the standard is GB/T11758-89-bee pollen. Single pollen rates are over 95%, and the production date was November 10, 2015.

### 2.2. Animal Studies

All animal experiments were performed using male ICR strain mice on a 12 h light/dark cycle in a pathogen-free animal feeding facility at Zhejiang Academy of Traditional Chinese Medicine. The animal study protocols were approved by the Animal Care and Use Committee of Zhejiang Academy of Traditional Chinese Medicine. At 6 weeks of age (weighted 26.37 ± 2.7 g), each mouse was fed rapeseed bee pollen (10 g/kg) by gavage. After a fixed time interval (3 h or 6 h on d1, d4, or d8), serum about 200 *μ*L was collected from each mouse, and then total RNA was extracted using mirVana*™* PARIS*™* Kit (AM1556, Ambion*™*).

### 2.3. Illumina Hiseq2500 Sequencing

The sequencing procedure was conducted according to standard steps provided by Illumina company, Inc. Briefly, a pair of adaptors were ligated to the 3′ and 5′ ends of total RNA. Reverse transcription followed by PCR is used to create cDNA constructs based on the small RNA ligated with 3′ and 5′ adapters. This process selectively enriches those fragments that have adapter molecules on both ends. Then the fragments of around 147–157 bp (22–30 nt length small RNA + adaptors) were purified by PAGE. The purified DNA was directly used for the cluster generation and sequencing using Illumina Hiseq2500 according to the manufacturer's instructions. The image files generated by the sequencer were then processed to produce digital data. The subsequent procedures included removing adapter dimers, junk, low complexity, common RNA families (rRNA, tRNA, snRNA, and snoRNA), and repeats. Subsequently, unique sequences with length in 18–26 nucleotides were mapped onto all plant miRNA precursors in miRBase 20.0 by BLAST search to identify known miRNAs and novel 3p- and 5p-derived miRNAs. Length variation at both 3′ and 5′ ends and one mismatch inside of the sequence were allowed in the alignment. The unique sequences mapping onto specific species mature miRNAs in hairpin arms were identified as known miRNAs. The unique sequences mapping onto the other arm of known specific species precursor hairpin opposite to the annotated mature miRNA-containing arm were considered to be novel 5p- or 3p-derived miRNA candidates.

### 2.4. Analysis of Level of miRNAs in Rapeseed Bee Pollen by RT-qPCR

Total RNA was extracted from 80 mg rapeseed bee pollen using Trizol (Invitrogen, Carlsbad, CA, USA) according to the manufacturer's protocol. Quantitative RT-PCR was performed using Taqman miRNA probes (Applied Biosystems, Foster City, CA, USA) according to the manufacturer's instructions. To calculate the absolute expression levels of target miRNAs, a series of synthetic miRNA oligonucleotides at known concentrations were reverse transcribed and amplified. The absolute amount of each miRNA was then calculated with reference to the standard curve. Quantitative PCR was performed using an ABI-StepOnePlus machine (Applied Biosystems).

### 2.5. Statistical Analysis

Differences are considered statistically significant at *P* < 0.05, using Student's* t*-test.

## 3. Results 

### 3.1. Raw Data Filtering

We sequenced a small RNA library from blood RNA of mouse fed with rapeseed bee pollen using the Illumina Hiseq2500 system. We acquired a total of 11,089,480 raw sequences. Overview of these reads from raw data to cleaned sequences is shown in Table 1.

**Figure 1:**
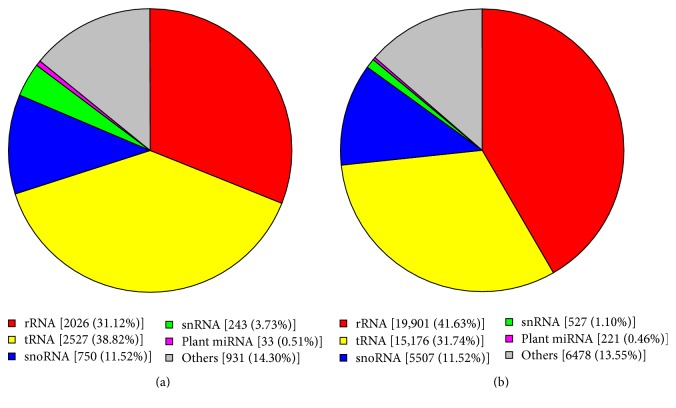
Pie chart of sequence category. (a) Pie chart of sequence category of total reads. (b) Pie chart of sequence category of unique reads.

We illustrated small RNA reads with Rfam dataset; to remove rRNA, scRNA, snoRNA, snRNA, and tRNA, the pie charts were drawn for total reads and unique reads (Figure 1).

### 3.2. Plant miRNAs Spectrum in Serum of Mice Fed with Rapeseed Bee Pollen

After removing the junk reads, the clean reads yield 34 plant miRNAs (Table 2). Plant miRNAs are 2′-O-methyl modified on their terminal nucleotide; in contrast, mammalian miRNAs with free 2′ and 3′ hydroxyls render plant miRNAs more difficult to be ligated to the cloning adapter compared with mammalian miRNAs. As a result, in the 11,089,480 raw reads, there were only 132 reads of plant miRNAs. However, some plant miRNAs can be detected even though the mammalian miRNAs caused a strong disturbance; in turn this proves that the content of plant miRNAs in mouse blood was not low. Among the plant miRNAs, miR-166a and miR-159 were with the highest levels in mouse blood; besides, these two miRNAs were both mapped onto rapeseed genome.

### 3.3. Comparison of Abundance Levels of miR-166a and miR-159 in Rapeseed Bee Pollen

Based on the predominant two miRNAs (miR-166a and miR-159) in the blood, we assumed that miR-166a and miR-159 can be found in rapeseed bee pollen, and the content of miRNAs in the rapeseed pollen also will follow the trend in the serum. To confirm this, the levels of miR-166a and miR-159 in rapeseed bee pollen were assessed by stem-loop quantitative reverse transcription polymerase chain reaction (RT-qPCR) assay. As a result, miR-166a and miR-159 can be detected in RNA of rapeseed bee pollen (Additional Figure 1 in Supplementary Material available online at http://dx.doi.org/10.1155/2016/5413849). Moreover, the CT values of miR-166a and miR-159 were 23.8 ± 0.23 and 31.22 ± 0.33, respectively, suggesting that the abundance level of miR-166a was higher than miR-159. These results suggested that food-derived exogenous miRNA from rapeseed bee pollen could be absorbed by mouse, and the abundance of specific miRNAs is dependent on their origins from the rapeseed bee pollen.

### 3.4. Comparison of Abundance Levels of miR-166a between Mice Fed with Rapeseed Bee Pollen and Control

**Figure 2:**
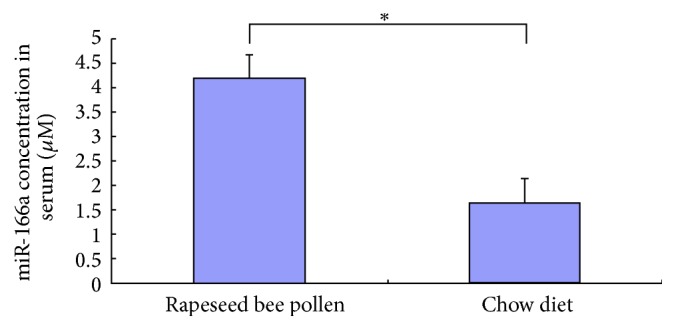
The abundance levels of miR-166a in mouse serum after feeding with rapeseed bee pollen or chow diet for 6 h (*n* = 5). ^*∗*^ *p* < 0.05.

Given that miR-166a is the highest abundance rape-encoded miRNA in mice fed with rapeseed bee pollen, and it is rich in rapeseed bee pollen, we speculate that the miR-166a in mouse serum are mainly absorbed from rapeseed bee pollen. To test this speculation, we compared the abundance level of miR-166a in serum of mice fed with rapeseed bee pollen and control. As it is reported that the levels of plant-based miRNAs were elevated in serum of mice for 6 h [4], we compared the levels of miR-166a in serum of mice fed with rapeseed bee pollen after 6 h and control. As shown in Figure 2 and additional Figure 2, the levels of miR-166a were elevated in serum of mice fed with rapeseed bee pollen for 6 h compared with control by RT-qPCR.

## 4. Discussion

An estimated 60% of all protein-coding genes are targeted by miRNAs in human [17]. In addition, many miRNAs are deregulated in immune system, inducing diseases like autoimmune diseases, inflammation, and tumors [3]. Food-derived miRNAs have the potential to restore the downregulated miRNAs in diseases. For example, immune-related miRNAs are abundant in breast milk, and they might play a critical role in the development of the infant immune system [18]. Furthermore, Western donor sera contained the plant miRNA miR159, whose abundance in the serum was inversely correlated with breast cancer incidence and progression in patients [9]. These studies raise the intriguing prospect of using edible plants miRNAs to prevent and treat mammal diseases.

Bee pollen is rich in nutrition and medicinal composition, which ensued a wide use of bee pollen in food, health products, medicine, cosmetics, and other fields [13, 19, 20]. In the field of medicals, bee pollen is used for prevention and treatment of prostate diseases [14], cardiovascular and cerebrovascular diseases, immune diseases, and so forth [15]. Besides, bee pollen is rich in RNA with a range of 0.6%–1% (w/w) [16].

In this study, we confirmed that miRNAs from rapeseed bee pollen can be absorbed by mice, and the abundance of exogenous miRNAs in mouse blood is dependent on their original levels in pollen. Moreover, the detailed functions of these exogenous miRNAs in mammals should be investigated to help clarify the immune function or medical efficacy of bee pollen. Nevertheless, the present study provided first hand evidence for the potential usages of rapeseed bee pollen as a supplement of plant miRNAs.

## Supplementary Material

The amplification curve of miR-159 (red) and miR-166a (green) by qPCR

## Figures and Tables

**Table 1:** Overview of reads.

Lib	Type	Total	% of total	Unique	% of unique
Raw reads	Nuclear acid	11,089,480	100.000	209,873	100.000
3ADT & length filter		320,992	2.895	145,808	69.474
Junk reads		858	0.008	610	0.291
Rfam	RNA	47,589	0.429	6,477	3.086
mRNA	RNA	3,694	0.033	905	0.431
Repeats	RNA	369	0.003	74	0.035
rRNA	RNA	19,901	0.179	2,026	0.965
tRNA	RNA	15,176	0.137	2,527	1.204
snoRNA	RNA	5,507	0.050	750	0.357
snRNA	RNA	527	0.005	243	0.116
Plant miRNA	RNA	221	0.002	33	0.016
Another Rfam RNA	RNA	6,478	0.058	931	0.444
Clean reads		10,716,785	96.639	56,136	26.748

**Table 2:** Plant miRNAs in mice fed with rapeseed bee pollen.

miRNA ID	miRNA sequence	Length (nt)	Frequency
bna-miR-166a	TCGGACCAGGCTTCATTCCCC	21	35
bna-miR-159	TTTGGATTGAAGGGAGCTCTA	21	22
gma-miR6300	GTCGTTGTAGTATAGTGGT	19	8
nta-miR6145e	ATTGTTACATGTAGCACTGGCT	22	7
nta-miR6146b	TTTGTCCAATGAAATACTTATC	22	6
nta-miR6020b	AAATGTTCTTCGAGTATCTTC	21	5
nta-miR6149a	TTGATACGCACCTGAATCGGC	21	5
ath-miR-166a	TTCGGACCAGGCTTCATTCCCC	22	3
osa-miR530	TGCATTTGCACCTGCACCTCC	21	3
ahy-miR408	TGCACTGCCTCTTCCCTGGCT	21	3
mdm-miR408a	TGCACTGCCTCTTCCCTGGCT	21	3
bna-miR397a	ATTGAGTGCAGCGTTGATG	19	2
peu-MIR2916	CAACCATAAACGATGCCGACCAGG	24	2
nta-miR168a	TCGCTTGGTGCAGGTCGGGAC	21	2
gma-miR482b	TCTTCCCTACACCTCCCATACC	22	2
nta-miR482a	TTTCCAATTCCACCCATTCCTA	22	2
nta-miR827	TTAGATGAACATCAACAAACA	21	2
ppt-miR894	TTCACGTCGGGTTCACCA	18	2
gma-miR3522	TGAGACCAAATGAGCAGCTGA	21	2
gma-miR4996	TAGAAGCTCCCCATGTTCTCA	21	2
bna-miR403	TTAGATTCACGCACAAACTCG	21	1
peu-MIR2916	ACCGTCCTAGTCTCAACCATA	21	1
aau-miR162	TCGATAAACCTCTGCATCCAG	21	1
bdi-miR398a	TATGTTCTCAGGTCGCCCCTGT	22	1
gma-miR403a	TTAGATTCACGCACAAACTT	20	1
gma-miR1507a	TCTCATTCCATACATCGTCTGA	22	1
nta-miR6159	TAGCATAGAATTCTCGCACCTA	22	1
hbr-miR6173	GCTGTAAACGATGGATACT	19	1
ptc-miR6478	CCGACCTTAGCTCAGTTGGT	20	1
stu-miR7997c	TTGCTCGGATTCTTCAAAAAT	21	1
bna-miR156b	TTGACAGAAGATAGAGAGCAC	21	1
gma-miR166m	GCGGACCAGGCTTCATTCCCC	21	1
stu-miR399a	GGGCTACTCTCTATTGGCATG	21	1
bna-miR156a	TGACAGAAGAGAGTGAGCAC	20	1
