# Supplementary material for: Identification of Dietetically Absorbed Rapeseed (Brassica campestris L.) Bee Pollen MicroRNAs in Serum of Mice
Source: Biomed Res Int. 2016 Aug 15;2016:5413849. doi: 10.1155/2016/5413849 (PMC5002473; doi:10.1155/2016/5413849)
Supplement: Supplementary file 1 — The amplification curve of miR-159 (red) and miR-166a (green) by qPCR [file 5413849.f1.pdf]

groups	Abundance (uM)				
Rapeseed bee pollen group	3.87	4.50	2.92	2.15	7.44
Control group	1.10	2.37	1.61	1.50	1.54

Additional table 1 The abundance of miR-166a in mice in groups of rapeseed bee pollen or control

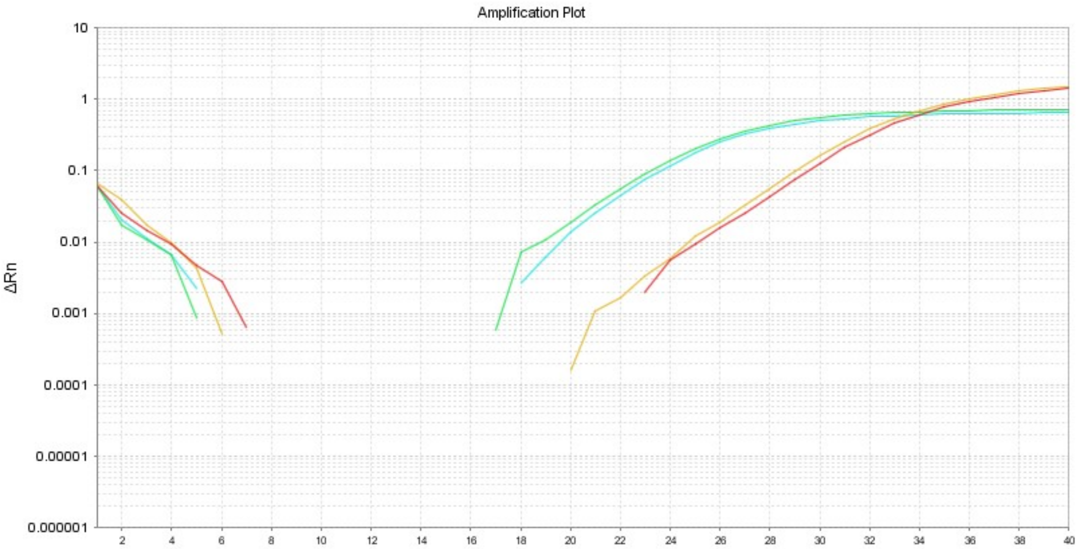

Additional Fig 1 The amplification curve of miR-159 (red) and miR-166a (green) by qPCR

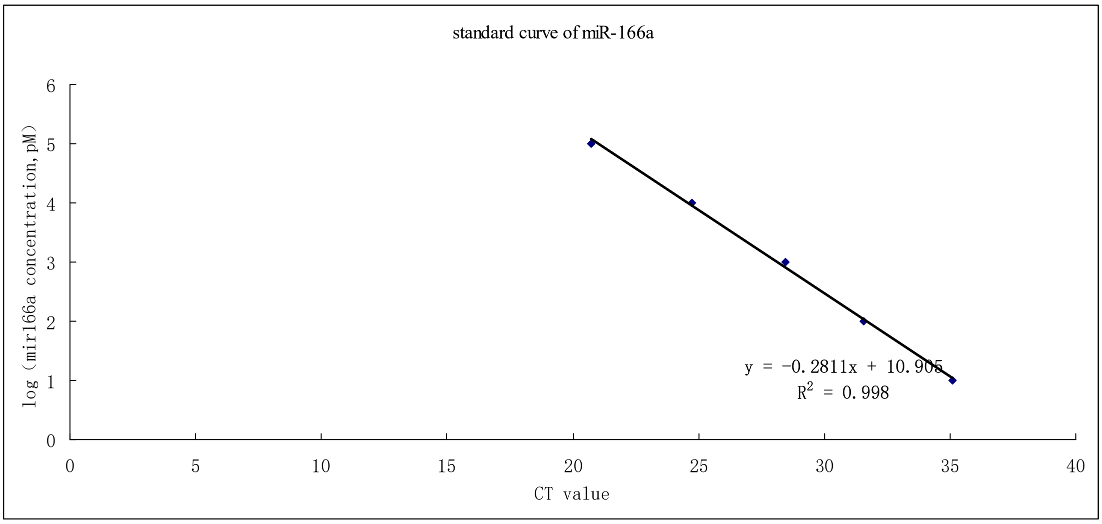

Additional Fig 2 Standard curve of miR-166a
